# Readout-segmented echo-planar diffusion-weighted MR for the evaluation of aggressive characteristics of rectal cancer

**DOI:** 10.1038/s41598-018-30488-5

**Published:** 2018-08-22

**Authors:** Chun-chao Xia, Jin Pu, Jin-ge Zhang, Wan-lin Peng, Lei Li, Fei Zhao, Kai Zhang, Yu-ming Li, Ke-ling Liu, Wen-jian Meng, Xiang-bing Deng, Xiao-yue Zhou, Zhen-lin Li

**Affiliations:** 1 Department of Radiology, West China Hospital, Sichuan University, 37# Guo Xue Xiang, Chengdu, Sichuan 610041 China; 2 Department of Gastrointestinal Surgery, West China Hospital, Sichuan University, 37# Guo Xue Xiang, Chengdu, Sichuan 610041 China; 3 Siemens Healthcare Ltd, Collaboration NEA, Shanghai, 200000 China

## Abstract

To evaluate whether aggressive characteristics of rectal cancer can be predicted by the apparent diffusion coefficient (ADC) obtained using readout-segmented echo-planar imaging (rs-EPI) diffusion-weighted magnetic resonance. We enrolled one hundred and fifteen patients. The image quality of ADC maps by rs-EPI was compared with that by traditional single-shot echo-planar imaging (ss-EPI), and ADC measurement was performed on the rs-EPI based ADC maps. Differences in ADC values of tumors grouped according to differentiation grade, clinical T stage and plasmatic carcinoembryonic antigen (CEA) level were tested. The correlation between each aggressive characteristic and the corresponding ADC values was evaluated. The image quality of ADC maps obtained by rs-EPI was superior toss-EPI (P < 0.05). The ADC values of tumor were categorized based on the following differentiation grades: poor (0.89 ± 0.12 × 10^−3^ mm^2^/s), moderate (1.13 ± 0.25 × 10^−3^ mm^2^/s), and good (1.31 ± 0.19 × 10^−3^ mm^2^/s); P < 0.001. Tumors with lower differentiation grades corresponded to lower ADC values (r = 0.59, P < 0.001). However, ADC differences were not observed in different clinical T stage (P = 0.22) and plasmatic CEA level (P = 0.38). Rs-EPI sequence-based ADC values represent a potential imaging marker for the aggressive rectal cancer characteristics.

## Introduction

Rectal cancer accounts for approximately 35% of colorectal cancers worldwide, and it has a worse prognosis than colon cancer because of its higher frequency of metastases and local recurrence^1^. Several factors, such as clinical staging, differentiation grade and carcinoembryonic antigen (CEA) levels, are associated with patient mortality, recurrence rate, and quality of life^2,3^. Therefore, imaging modalities that can provide detailed information are required for optimal treatment. In 2012, the European Society of Gastrointestinal and Abdominal Radiology reached a consensus that MRI is a suitable imaging modality for the noninvasive evaluation of rectal cancer before clinical decision-making^4^. In recent years, diffusion-weighted imaging (DWI), a functional MRI technique, has been applied for the evaluation of neoadjuvant-combined treatment responses in rectal cancer. Furthermore, reports have indicated that the apparent diffusion coefficient (ADC) value can differentiate benign and malignant lesions in the rectum^5–9^.

Readout-segmented echo-planar imaging (rs-EPI) diffusion-weighted imaging (DWI), in which k-space is segmented to shorten the echo spacing, exhibits reduced spatial distortion and improved resolution compared with other imaging approaches^10–13^. Our previous study demonstrated that rs-EPI improves the image quality of DWI in rectal patients compared with that using traditional approach of single-shot echo-planar imaging (ss-EPI)^14^. However, to the best of our knowledge, further studies have not reported the image quality of ADC maps for rectum derived from rs-EPI, and whether ADC values obtained by this newly introduced sequence are correlated with important aggressive characteristics of rectal cancer. Therefore, the purpose of this study was to evaluate the potential role of the ADC values obtained by rs-EPI in the prediction of aggressive characteristics of rectal cancer.

## Results

### Baseline characteristics

A total of 115 patients (77 males and 38 females, average age: 60.3 ± 11.5 years) underwent both ss-EPI and rs-EPI examination without complications. The majority of these patients had a moderate differentiation grade (92/115, 80.0%), whereas the remainder had a poor (11/115, 9.6%) or good (12/115, 10.4%) differentiation grade. Based on the MR findings, 9 patients (9/115, 7.8%) were in the T1 stage, 40 patients (40/115, 34.8%) were in the T2 stage, 53 patients (53/115, 46.1%) were in the T3 stage, and 13 patients (13/115, 11.3%) were in the T4 stage. In addition, 76 patients (76/115, 66.1%) had a CEA level of <5 ng/ml, whereas 39 patients (39/115, 33.9%) had a CEA level of ≥5 ng/ml.

### Image quality of ADC maps

**Figure 1:**
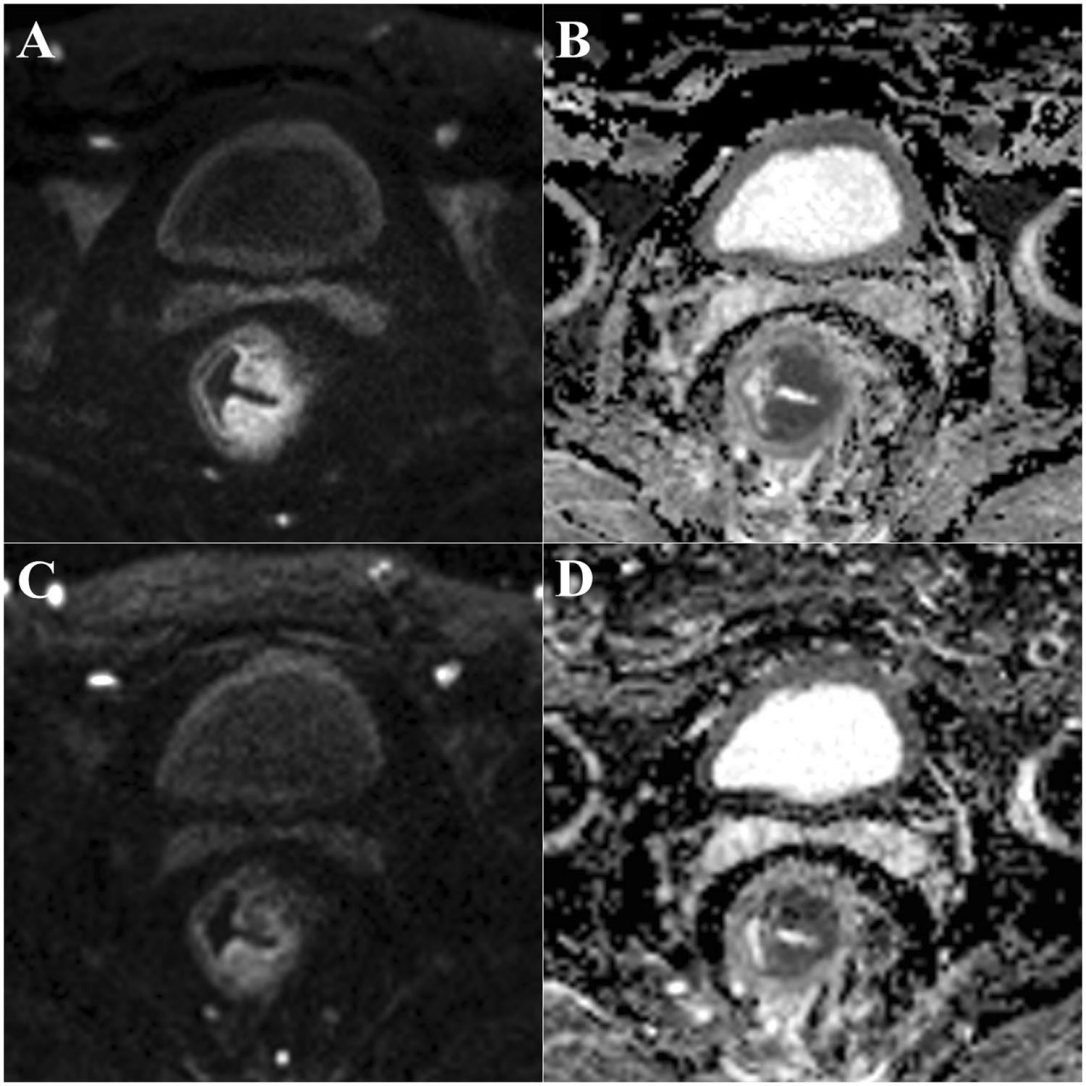
DW images and ADC maps obtained by rs-EPI (**A**,**B**) and ss-EPI (**C**,**D**) in the same patient with rectal cancer. Abbreviations: ADC, apparent diffusion coefficient; rs-EPI, readout-segmented echo-planar imaging; ss-EPI, single-shot echo-planar imaging.

The scores of the two independent readers were highly consistent (kappa values ranged from 0.82 to 0.88) for the ADC maps derived from rs-EPI sequence. More importantly, rs-EPI was superior to ss-EPI in the geometric distortion, spatial resolution, lesion conspicuity and comprehensive imaging quality, respectively (all P < 0.05) (Table 1; Fig. 1).

**Table 1:** Image quality of ADC maps obtained by rs –EPI and ss-EPI.

Parameters	rs-EPI			ss-EPI		
	Reader 1	Reader 2	Kappa	Reader 1	Reader 2	Kappa
Geometric distortion	4.84 ± 0.37*	4.83 ± 0.38*	0.83	4.29 ± 0.67	4.15 ± 0.57	0.80
Spatial resolution	4.79 ± 0.41*	4.77 ± 0.42*	0.82	3.84 ± 0.78	3.71 ± 0.62	0.71
Lesion conspicuity	4.76 ± 0.49*	4.72 ± 0.49*	0.87	3.56 ± 0.74	3.47 ± 0.76	0.70
Comprehensive image quality	4.79 ± 0.41*	4.81 ± 0.40*	0.88	3.72 ± 0.57	3.72 ± 0.59	0.85

Note: Values are presented as the mean ± SD.rs-EPI, readout-segmented echo-planar imaging. ss-EPI, single-shot echo-planar imaging. *rs-EPI vs. ss-EPI, P < 0.05.

### Correlations between tumor ADC values and aggressive characteristics

**Figure 2:**
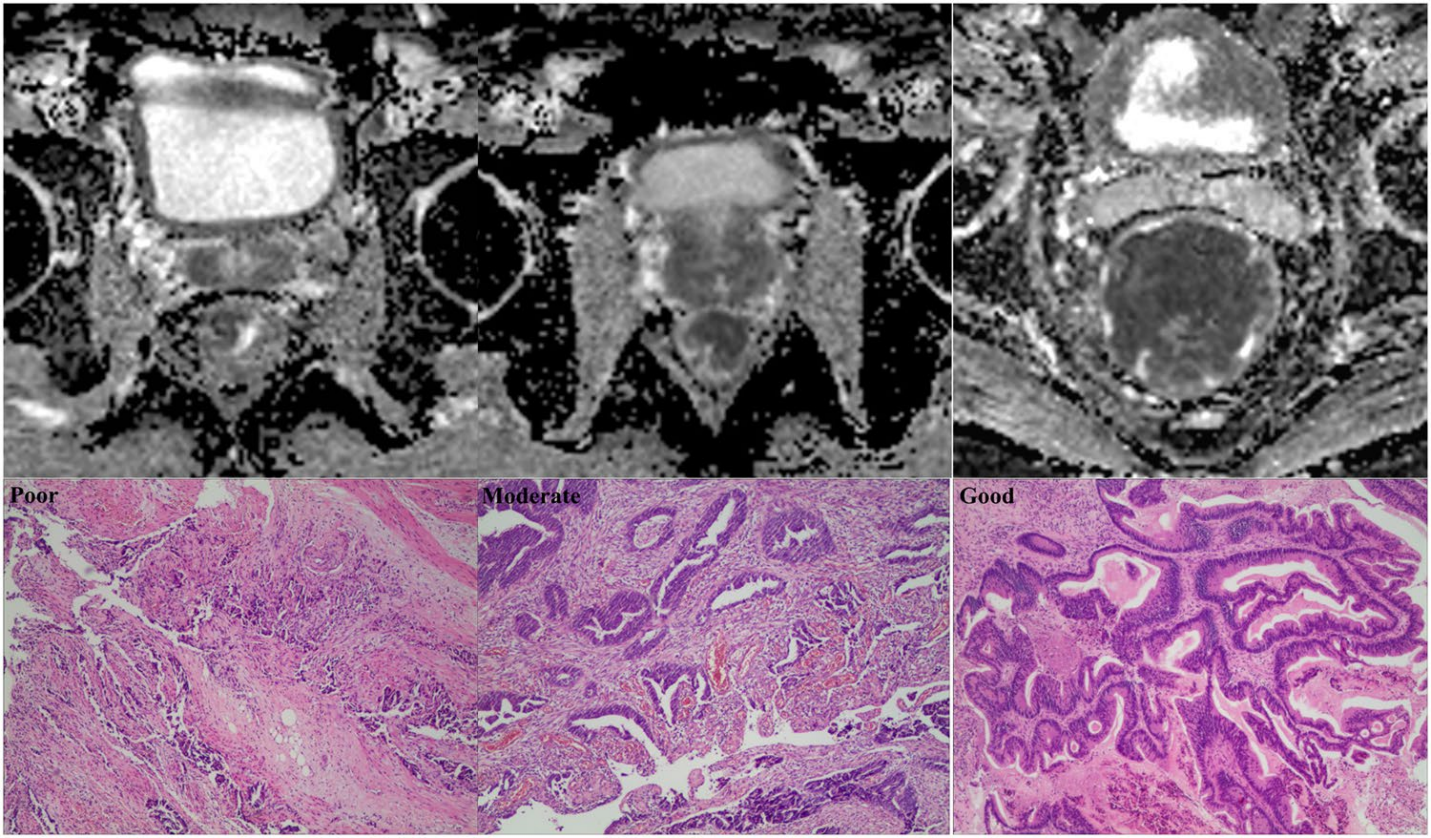
Different differentiation grade of tumors and corresponding ADC maps obtained by rs-EPI. Abbreviations as in Fig. 1.

**Figure 3:**
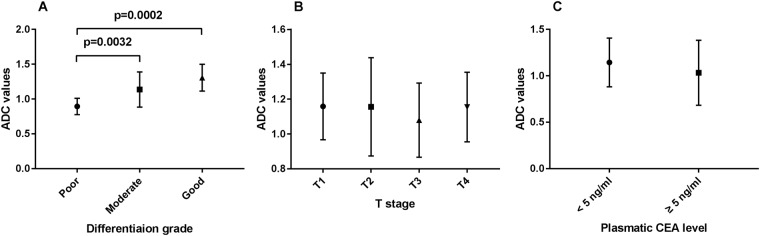
ADC values among different differentiation grade **(A)**, clinical T stage **(B)** and plasmatic CEA level **(C)**. Abbreviations: CEA, carcinoembryonic antigen.

**Figure 4:**
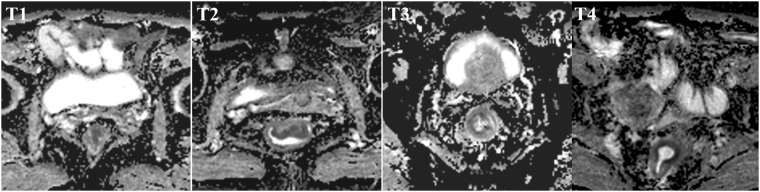
Different clinical T stage of tumors and corresponding ADC maps obtained by rs-EPI. Abbreviations as in Fig. 1.

**Figure 5:**
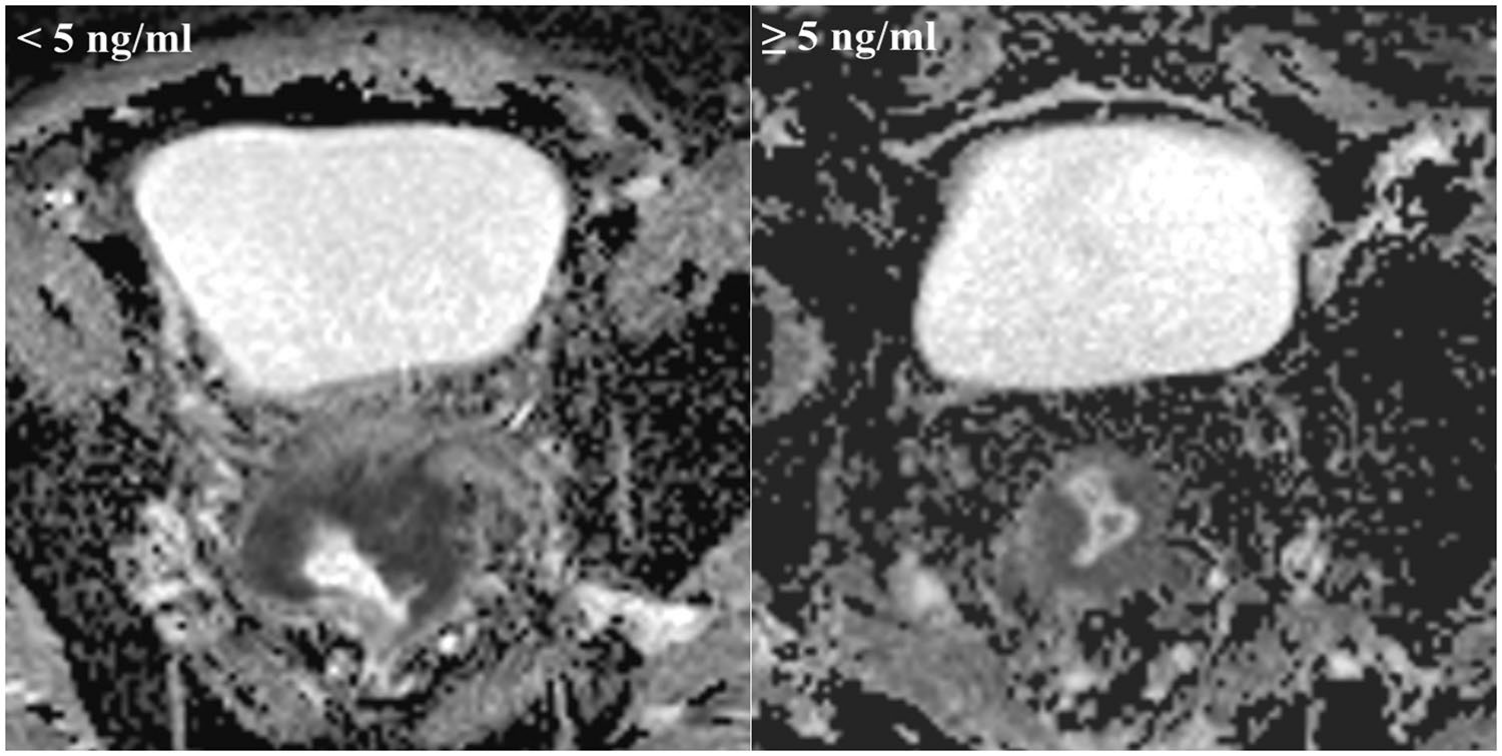
Plasmatic CEA level (low/high) and corresponding ADC maps obtained by rs-EPI. Abbreviations as in Figs 1 and 3.

The mean ADC values differed among the poor, moderate and good differentiation grade tumors (0.89 ± 0.12 × 10^−3^ mm^2^/s vs. 1.13 ± 0.25 × 10^−3^ mm^2^/s vs. 1.31 ± 0.19 × 10^−3^ mm^2^/s, respectively; P < 0.001) (Table 2; Figs 2, 3A). The mean ADC values of the tumors were not significantly different in each T stage (1.06 ± 0.08 × 10^−3^ mm^2^/s vs. 1.11 ± 0.20 × 10^−3^ mm^2^/s vs. 1.03 ± 0.18 × 10^−3^ mm^2^/s vs. 1.15 ± 0.18; P = 0.22) (Table 2; Figs 3B, 4). The mean ADC values of the tumors in patients with < 5 ng/ml CEA were not significantly different compared with those of patients with ≥ 5 ng/ml CEA (1.10 ± 0.25 × 10^−3^ mm^2^/s vs. 1.07 ± 0.19 × 10^−3^ mm^2^/s, respectively, P = 0.38) (Table 2; Figs 3C, 5).

**Table 2:** Correlations between the tumor ADC values and aggressive characteristics.

Parameters	No. of patients (n)	Tumor ADC values (×10^−3^ mm^2^/s)	Spearman’s coefficient
**Differentiation grade**			
Poor	11 (9.6%)	0.89 ± 0.12	0.59**
Moderate	92 (80.0%)	1.13 ± 0.25*	
Good	12 (10.4%)	1.31 ± 0.19*	
**Clinical T stage**			
T1	9 (7.8%)	1.06 ± 0.08	−0.08
T2	40 (34.8%)	1.11 ± 0.20	
T3	53 (46.1%)	1.03 ± 0.18	
T4	13 (11.3%)	1.15 ± 0.18	
**Plasma CEA level**			
<5 ng/ml	76 (66.1%)	1.10 ± 0.25	−0.07
≥5 ng/ml	39 (33.9%)	1.07 ± 0.19	

Notes: Values are presented as the mean ± SD. ADC, apparent diffusion coefficient; CEA, carcinoembryonic antigen. *P < 0.001 vs. poor differentiation grade group. **P < 0.001.

As shown in Table 2, Spearman’s rank test indicated a good positive correlation between the differentiation grade and ADC value (r = 0.59, P < 0.001) but a weak correlation between the clinical T stage (r = −0.08, P = 0.41) and CEA level (r = −0.07, P = 0.38), respectively.

### Inter-observer variability

**Figure 6:**
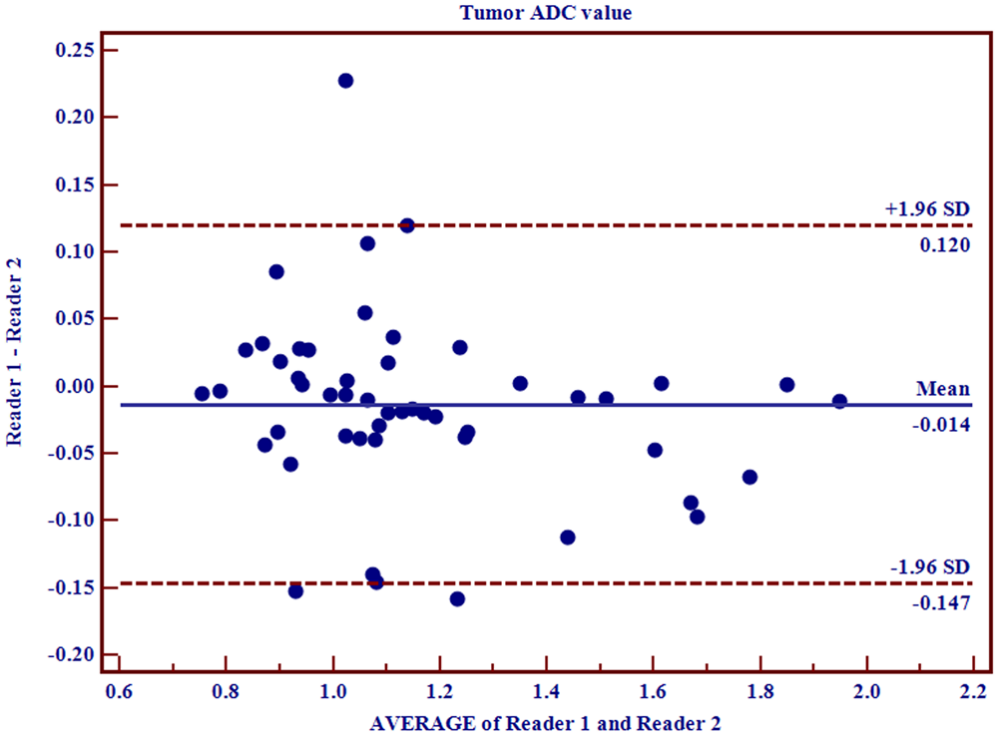
Bland-Altman plots showing the consistency between reader 1 and reader 2 (n = 50). The biases for the tumor ADC values were −0.014. Abbreviations as in Fig. 1.

Among the 115 patients, 50 of them were randomly selected for the inter-observer variability evaluations of the tumor ADC values. Bland-Altman plots demonstrated an acceptable level of bias as shown in Fig. 6.

## Discussion

Recent advances in the treatment of rectal cancer have shown that neoadjuvant-combined therapies may be beneficial for high-risk patients and have the potential to improve local control and survival^5^. MR techniques play a crucial role in the treatment when evaluating the clinical staging of rectal cancer and predicting responses after neoadjuvant chemotherapy^1,3–7^. Among these techniques, DWI serves as a promising method for the early identification of rectal cancer because it can differentiate between rectal tumor and normal tissue via the diffusion of water in these tissues^15^. Moreover, DWI is a noninvasive method that does not require a contrast agent; however, DW images acquired using the ss-EPI sequence suffer from low resolution and spatial distortion that may affect diagnostic accuracy^10,14^. Currently, rs-EPI represents an alternative sequence for DWI because of its improved resolution and reduced distortion compared with those of ss-EPI, and it has already been applied for brain, breast and liver tissue^12,13,16^. Our previous research revealed that DW images based on the rs-EPI sequence allows for the accurate recognition of rectal tumors^14^. Furthermore, according to the data presented in this study, the image quality of ADC maps derived from rs-EPI was superior to that of ss-EPI. The high-risk patients are assumed to gain benefit if ADC measurement was made on rs-EPI. Therefore, the relationship between ADC values obtained via this sequence and important aggressive characteristics of rectal cancer must be further clarified.

Our results indicated that tumors with lower differentiation grades corresponded to relatively lower mean ADC values (r = 0.59, P < 0.001), and a similar trend of ADC values has been observed in previous studies^2,17–19^. To date, the intrinsic association between DW MR imaging and histopathological findings at microscopic level remains unclear. One possible explanation for this phenomenon is the different cellular microarchitectures of rectal cancer. Tumor with poor differentiation grade appears hypointense on ADC maps. The underlying mechanism may be that the aggressive cell proliferation leads to a high cellular density and a decreased interstitial volume, which restricts the motion of the protons. On the contrary, tumor with good differentiation grade has a relatively low cellular density and a large interstitial space when compared with poor differentiation grade tumor. Therefore, this subgroup presents a hyperintense on ADC maps^20^. Further studies are needed to address this issue. Even so, the results suggest that rs-EPI-based ADC values have the potential for use as imaging markers for the assessment of aggressive characteristics of rectal cancer.

Significant differences were not observed among the ADC values for each clinical T stage subgroup. This finding was consistent with previous study reported by Attenberger *et al*., which suggested that ADC measurement should be quantified throughout the whole tumor rather than merely performed on a single slice (e.g. the center slice of the tumor) by manually-drawn ROI^21^. However, even took the heterogeneous nature of tumor into consideration and then measured the ADC values throughout the whole tumor volume, the results were not differing among clinical T stage. There were also some studies indicated that the ADC value had the potential to differentiate the T stage of a tumor, a consensus has not been reached in clinical practice^2,22^.

CEA is a plasmatic marker for rectal cancer, but with poor specificity. In our study, patients with low CEA level showed no statistical difference in ADC values compared with those with high CEA level.

Our study had several limitations. First, the ADC measurement was only reported for rs-EPI sequence. According to the results from our study, the image quality of rs-EPI sequence was superior to that of ss-EPI sequence. Hence, we deemed that the ADC value derived from rs-EPI sequence was more optimal for further analysis given that aim of this study was mainly focused on the relationship between the ADC value and aggressive characteristics. For this reason, the ADC measurement was only performed on rs-EPI images. Second, although lymph node metastasis is another important aggressive characteristic of rectal cancer, the evaluation of lymph nodes via rs-EPI was not included in this study. Such evaluations will be discussed in a future study.

In conclusion, our study demonstrated that rs-EPI-based ADC values tend to decrease as the differentiation grade decreases. Therefore, ADC values have the potential for use as an imaging marker for the assessment of aggressive characteristics of rectal cancer.

## Materials and Methods

### Study population

From October 2015 to October 2016, one hundred and fifteen consecutive patients who were referred to our hospital were enrolled in this study. Among these patients, there were a total of ten patients that were included in our previous work. The inclusion criterion was biopsy-proven rectal cancer either by endoscopically guided biopsy or surgical resection. Those patients with radiochemotherapy treatment prior to magnetic resonance (MR) examination and incomplete clinical data were not recruited for this study. The institutional review board of West China hospital approved this study. Written informed consent was obtained from all patients. All patient-sensitive information was treated with full confidentiality and used solely for the purpose of this study.

### MR protocol

Scanning was performed with a 3.0 T MRI scanner (Magnetom Skyra, Siemens Medical Solutions, Erlangen, Germany) with an 18-channel soft coil. DW images were acquired using two sequences (ss-EPI and rs-EPI) and subsequently used for ADC maps reconstruction. The scanning parameters of ss-EPI were as follows: TE = 88 msec, TR = 5000 msec, TI = 210 msec, slice thickness = 4.5 mm, field of view = 216 × 216 mm, matrix size = 128 × 128, b values = 0 and 1000 s/mm^2^, intersection gap = 10%, echo spacing = 0.94 msec, in-plane resolution = 1.7 × 1.7 mm, acquisition time = 1:05 min:s, No. of readout segment = 3, fat suppression = fat sat. Strong. Frequency selected pulse followed by a spoiled gradient was used. The scanning parameters of rs-EPI were as follows: TE = 66 msec, TR = 5000 msec, TI = 210 msec, slice thickness = 4.5 mm, field of view = 216 × 216 mm, matrix size = 160 × 160, b values = 0 and 1000 s/mm^2^, intersection gap = 10%, echo spacing = 0.4 msec, in-plane resolution = 1.4 × 1.4 mm, acquisition time = 5:17 min:s, No. of readout segment = 3, fat suppression = fat sat. Strong. Frequency selected pulse followed by a spoiled gradient was used. Intravenous contrast was not applied in this study.

### Image analysis

All databases analyses were performed on a workstation (Syngo; Siemens Medical System, Forchheim, Germany). Image quality of ADC maps obtained by those two sequences was evaluated using a five-point scale that assessed the geometric distortion, spatial resolution, lesion conspicuity, and comprehensive image quality. A score of five = images with excellent anatomic details that were free of artifacts; a score of four = images with slight artifacts that were not affected the visualization of anatomic details; a score of three = images with moderate artifacts that were not affected the ADC measurement; a score of two = images with moderate artifacts that will affected the ADC measurement; and a score of one was assigned to images that displayed severe artifacts and did not provide useful, clinically required anatomical information. A score of 4 to 5 was regarded as satisfactory. Two experienced radiologists (reader 1 and reader 2), who were blind to the types of MR sequence, the histopathologic results as well as the score of the other radiologist, participated in evaluating image quality.

The tumor ADC measurement was performed on the ADC maps derived from rs-EPI by the same two radiologists. The region of interest (ROI) was outlined manually along the edge of the tumor section by section at a slice thickness of 4.5 mm on DW MR images with a b value of 1000 sec/mm^2^. The size of ROI in each section was no less than 10 voxels. The ROIs were transferred onto ADC maps after drawing in all imaging sections. The mean ADC values of each tumor were acquired from the mean of each ROI by using O. K software (GE Healthcare, Shanghai, China)^14^.

The inter-observer variability for ADC measurement was determined by comparison the results in randomized 50 cases.

### Aggressive characteristics

The differentiation grade (poor, moderate and good) of each patient was determined by an experienced pathologist who was unaware of the tumor ADC measurement. The clinical staging of rectal cancer was evaluated via MR examination based on the 7^th^ edition of the cancer staging manual of American Joint Committee on Cancer. The plasmatic CEA level was established prior to endoscopic-guided biopsy or surgical resection.

### Statistical analysis

Statistical analyses were performed with GraphPad Software (Version 7.0; GraphPad Software, Inc., San Diego, California, USA) and MedCalc software (version 15.6, MedCalc software, Mariakerke, Belgium). D′Agostino-Pearson normality test was used to check the normality of data, and values were expressed as mean ± standard deviations or median ± interquartile range. The kappa test was used to assess the consistency between the five-point scale grades of image quality obtained by the two radiologists. Kappa values over 0.75, from 0.75 to 0.4, and below 0.4 were considered excellent, good to fair, and poor, respectively. Wilcoxon rank test was used to compare the scores of ADC maps between the two sequences. Mann-Whitney test was used to compare the differences in tumor ADC values between the following two groups: patients with CEA < 5 ng/ml and patients with CEA ≥5 ng/ml. One-way analysis of variance was used to compare the differences in tumor ADC values among the groups with different differentiation grades. Kruskal-Wallis test was used to compare the differences in tumor ADC values among clinical T stages. Spearman’s rank test was used to determine the correlation between each aggressive characteristic and the corresponding ADC values. Bland-Altman analysis was used to assess the inter-observer variability in tumor ADC measurement. A two-tailed P value of <0.05 was considered statistically significant in all analyses.

### “Compliance with ethical standards”

This study involving human participants was approved by the Institutional Review Board (IRB) of West China hospital, and we pledged to abide by the declaration of Helsinki (2000 EDITION) in accordance with the relevant medical research rules of China in the study. Written informed consent was obtained from all patients. All patient-sensitive information was treated with full confidentiality and used solely for the purpose of this study.

## Data Availability

The datasets generated and analyzed during the current study are available from the corresponding author on reasonable request.
